# Visual agnosia and prosopagnosia secondary to melanoma metastases: case report

**DOI:** 10.1590/S1980-57642008DN10100016

**Published:** 2007

**Authors:** Norberto Anízio Ferreira Frota, Lécio Figueira Pinto, Claudia Sellitto Porto, Paulo Henrique Pires de Aguia, Luiz Henrique Martins Castro, Paulo Caramelli

**Affiliations:** 1 MD, Department of Neurology, University of São Paulo School of Medicine, Behavioral and Cognitive Neurology Unit Fellow.; 2 MD, Department of Neurology, University of São Paulo School of Medicine, Electroencephalography-Epilepsy Fellow;; 3 MD, PhD, Behavioral and Cognitive Neurology Unit Psychology.; 4 MD, PhD, Department of Neurosurgery, University of São Paulo School of Medicine;; 5 MD, PhD, Department of Neurology, University of São Paulo School of Medicine;; 6 MD, PhD, Behavioral and Cognitive Neurology Unit Faculty of Medicine, Federal University of Minas Gerais.

**Keywords:** prosopagnosia, visual agnosia, metastasis, melanoma, prosopagnosia, agnosia visual, metastase, melanoma

## Abstract

The association of visual agnosia and prosopagnosia with cerebral metastasis is very rare. The presence of symmetric and bilateral cerebral metastases of melanoma is also uncommon.We report the case of a 34 year-old man who was admitted to hospital with seizures and a three-month history of headache, with blurred vision during the past month. A previous history of melanoma resection was obtained. CT of the skull showed bilateral heterogeneous hypodense lesions in the occipito-temporal regions, with a ring pattern of contrast enhancement. Surgical resection of both metastatic lesions was performed after which the patient developed visual agnosia and prosopagnosia. On follow-up, he showed partial recovery of visual agnosia, while prosopagnosia was still evident. The relevance of this case is the rare presentation of metastatic malignant melanoma affecting homologous occipito-temporal areas associated with prosopagnosia and associative visual agnosia.

## Abstract (pt)

A associação de agnosia visual e prosopagnosia com metástase cerebral são muito raras. A presença de lesões metastáticas de melanoma, simétricas e bilaterais, também é incomum. Nós descrevemos o caso de um homem de 34 anos que foi admitido no hospital com convulsões e uma história de cefaléia há três meses, com dificuldade visual no último mês. História pregressa revelava ressecção de melanoma. TC de crânio evidenciou lesões hipodensas, heterogêneas, bilaterais nas regiões occipito-temporais, com captação de contraste anelar. A ressecção cirúrgica de ambas as metástases cerebrais foi realizada e o paciente desenvolveu agnosia visual e prosopagnosia após o procedimento. Na evolução houve melhora parcial da agnosia visual enquanto a prosopagnosia ainda estava evidente. A relevância deste caso consiste na apresentação rara de melanoma maligno metastático afetando as áreas occipito-temporais homólogas associada com prosopagnosia e agnosia visual associativa.

Visual agnosia is defined as a disorder of the visual process leading to dysfunction in perception and/or recognition of objects, faces (prosopagnosia), letters (alexia) or colors (achromatopsia), either in isolation or combination^1-4^.

Prosopagnosia was defined by Bodamer in 1947, although it had been known since the XIX century, according to a description by Quaglino, Hughlings Jackson and Charcot. It is associated with bilateral lesions in the fusiform gyrus, although unilateral lesions involving the right fusiform gyrus have also been described^5^.

Ischemic lesions are the most frequent causes, but focal atrophy, congenital disorders and tumors can also be associated with prosopagnosia^1^.

Brain metastases are the most frequently encountered intracranial tumors. Malignant melanoma is the third most frequently encountered histologic type of brain metastasis. Occipital lobe lesions represent 5.5% of brain metastasis^5,6^. Association with prosopagnosia has previously been described in only one patient^7^.

## Case report

A 34 year-old man, a professional cook, with an educational level of four years, presented a clinical history of headache, nausea and vomiting in the past three months. He also complained of a visual disorder described as a “reading difficulty” in the last month.

The patient was admitted to the emergency room because of a tonic-clonic seizure the day before. On examination, the patient was confused and somnolent, but arousable, with reactive pupils and no motor deficits.

**Figure 1:**
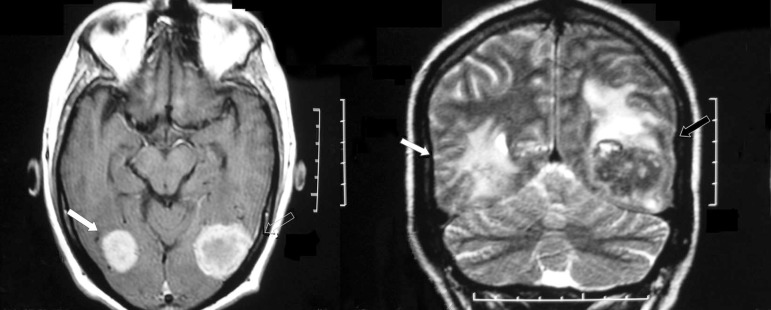
Preoperative MRI: arrows showing bilateral occipito-temporal metastasis with contrast enhancement.

The magnetic resonance imaging (MRI) showed bilateral occipito-temporal lesions suggestive of brain metastasis. These lesions, involved the medial and inferior occipital gyrus bilaterally, the left fusiform and lingual gyrus and part of the right fusiform gyrus (Figure 1). Eight years before, the patient had undergone partial resection of a malignant melanoma in the nose, without further treatment.

Subsequently, he was submitted to a neurosurgical procedure involving total metastasis resection through occipital craniectomy. A ZEISS 588 microscope was used to access both lesions. Following surgery, the patient was admitted to the critical care unit.

After surgery, the patient had no motor, sensory or coordination deficits, but presented associative visual agnosia for colors, objects, faces and letters.

**Figure 2:**
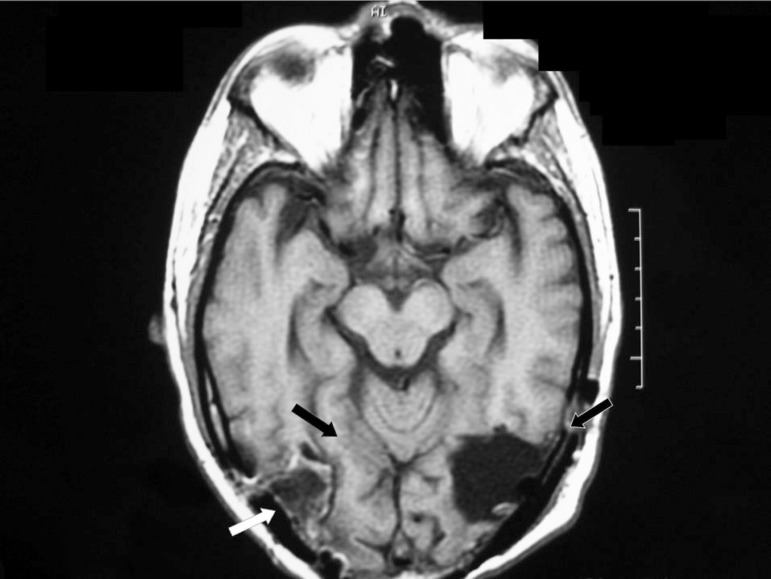
Postoperative MRI: To the right, white arrows showing involvement of inferior and occipital gyrus, with black arrows showing relative preservation of the right fusiform gyrus. To the left the black arrows show involvement of inferior and medial occipital gyrus and fusiform.

Upon hospital discharge, he underwent whole-head radiotherapy with 30 Gy, followed by chemotherapy using dacarbazine. On follow-up the MRI was repeated, showing no signs of residual brain metastasis (Figure 2).

The patient was re-evaluated at the outpatient unit 30 days after surgery. He complained of difficulties in recognizing faces.

On neurological examination, he scored 21 out of 30 points on the Mini Mental State Examination (MMSE). No motor, sensory or coordination deficits were recorded. The examination of the cranial nerves was unremarkable. Visual recognition deficits were still evident, albeit improved since surgery. Neuropsychological testing three months after surgery confirmed visual recognition deficits, more markedly for faces and colors (Table 1).

**Table 1:** Neuropsychological testing: face and color discrimination.

	Test	Patient
Visual function	Thematic figure	Severe difficulty in describing picture parts
	Raven progressive matrices	Color series: 16/36 *(10thpercentile)*
	Face recognition	Familiar faces (12 stimuli): no recognition;
		Famous faces (4 stimuli): recognition of one face (after prompt)

Table 2 shows impaired performance in other neuropsychological tests.

**Table 2:** Neuropsychological testing: attention, executive functions, memory, language and visuospatial skills.

Visuospatial skills	Block design (WAIS)	Severe impairment
	Rey-Osterrieth complex figure	Severe impairment
Attention	Trail making test	Unable to perform test
	Stroop test	Unable to recognize colors
Planning	Chapuis labyrinth	Mild impairment
	Wisconsin card sorting test	Number of errors = 32/64
		Number of categories = 01/03
		Numbers of perseveration errors = 15
Memory	Wechsler memory scale-	Logical memory (immediate recall) = normal
	review (WMS-R)	Visual reproduction (immediate recall) = 21/41 *(4^th^percentile)*
		Delayed recall
		Logical memory = normal
		Visual reproduction =10/41 *(1^st^percentile)*
	Rey-Osterrieth complex figure	Delayed recall = 06/36 (moderate impairment )
Language	Verbal fluency	Semantic category (animals): normal performance
		Phonemic category (FA.S): normal performance
	Boston naming test	10/60

The patient died nine months after surgery due to multiple metastases (liver, spleen, skin and bones).

## Discussion

The pattern of distribution of the lesions involving, bilaterally and almost symmetrically, the medial and inferior occipital gyrus, the left fusiform and lingual gyrus and part of the right fusiform gyrus is unusual, especially for brain metastasis. These areas play a critical role in visual recognition of objects, faces, colors and letters and are known as the “what system” of the central visual processing^1^.

Deficits presented soon after surgery were more obvious, probably due to brain swelling, surgical manipulation and brain resection, characterizing a clinical picture of associative visual agnosia. The patient underwent neuropsychological testing approximately three months postoperatively (Tables 1 and 2) which revealed below-average scores in almost all cognitive domains, except episodic memory and verbal fluency.Visual agnosia had become more prominent and might have influenced performance in the other tests. Recognition for faces and colors was more affected than for objects.

Prosopagnosia was initially associated with bilateral occipito-temporal lesions, most of them being ischemic^1-3^. Right fusiform gyrus lesions are also described in association to prosopagnosia^5,9^. Degenerative, vascular, traumatic and congenital lesions are possible etiologies^1^, but a metastatic lesion has been described in one case^8^.

Achromatopsy is also associated with occipito-temporal lesions, mainly on the right side. In the majority of cases it has a favorable outcome, except when the lesion is more posteriorly located, the case in our patient^4^.

In this patient, the right fusiform gyrus involvement was partial, but the medial and inferior occipital gyri were also involved. This pattern of anatomical distribution of the lesions probably disrupted the visual processing network for face recognition, as described by Rossion^10^.

The relevance of the present case is justified not only because of the rarity of prosopagnosia due to brain metastasis, but also to the relative preservation of the right fusiform gyrus, indicating the importance of other areas in the visual process related to face recognition.
